# Alcohol consumption and neurocognitive deficits in people with well-treated HIV in Switzerland

**DOI:** 10.1371/journal.pone.0246579

**Published:** 2021-03-02

**Authors:** Katharine E. A. Darling, Isabella Locatelli, Nadia Benghalem, Isaure Nadin, Alexandra Calmy, Klemens Gutbrod, Christoph Hauser, Peter Brugger, Barbara Hasse, Helen Kovari, Ursi Kunze, Marcel Stoeckle, Christophe Fux, Stefania Rossi, Caroline Di Benedetto, Severin Früh, Patrick Schmid, Philip E. Tarr, Jean-Bernard Daeppen, Renaud Du Pasquier, Matthias Cavassini, on behalf of the NAMACO Study Group, Swiss HIV Cohort Study

**Affiliations:** 1 Infectious Diseases Service, Lausanne University Hospital, Lausanne, Switzerland; 2 Division of Biostatistics and Quantitative Methods, Institute of Social and Preventive Medicine, Lausanne University Hospital, Lausanne, Switzerland; 3 Department of Clinical Neurosciences, Laboratory of Neuroimmunology, Research Centre of Clinical Neurosciences, Lausanne University Hospital, Lausanne, Switzerland; 4 HIV Unit, Infectious Diseases Division, Medicine Specialties Department, Geneva University Hospital, University of Geneva, Geneva, Switzerland; 5 Division of Cognitive and Restorative Neurology, Department of Neurology, Inselspital Bern, Bern, Switzerland; 6 Department of Infectious Diseases, Bern University Hospital, University of Bern, Bern, Switzerland; 7 Department of Neuropsychology, Neurology Clinic, University Hospital Zürich, Zürich, Switzerland; 8 Department of Infectious Diseases and Hospital Epidemiology, Universitätsspital Zurich, University of Zurich, Zurich, Switzerland; 9 Memory Clinic, Felix Platter Hospital, University Centre for Medicine of Aging, Basel, Switzerland; 10 Division of Infectious Diseases and Hospital Epidemiology, University Hospital Basel, University of Basel, Basel, Switzerland; 11 Infectious Diseases and Hospital Epidemiology Department, Kantonsspital Aarau, Aarau, Switzerland; 12 Neuropsychology Unit, Lugano Regional Hospital, Lugano, Switzerland; 13 Infectious Diseases Division, Lugano Regional Hospital, Lugano, Switzerland; 14 Neuropsychology Unit, Department of Neurology, Kantonsspital St. Gallen, St. Gallen, Switzerland; 15 Infectious Diseases and Hospital Epidemiology Division, Kantonsspital St. Gallen, St. Gallen, Switzerland; 16 University Department of Medicine, Kantonsspital Bruderholz, University of Basel, Bruderholz, Switzerland; 17 Service of Addiction Medicine, Lausanne University Hospital, Lausanne, Switzerland; 18 Service of Neurology, Department of clinical neurosciences, Lausanne University Hospital, Lausanne, Switzerland; The University of New South Wales, Neuroscience Research Australia, AUSTRALIA

## Abstract

**Background:**

Hazardous alcohol consumption and HIV infection increase the risk of neurocognitive impairment (NCI). We examined the association between alcohol consumption and specific neurocognitive domain function in people with HIV (PWH) taking modern antiretroviral therapy.

**Methods:**

The Neurocognitive Assessment in the Metabolic and Aging Cohort (NAMACO) study is a prospective, longitudinal, multicentre and multilingual (French, German and Italian) study of patients aged ≥45 years old enrolled in the Swiss HIV Cohort Study (SHCS). Baseline data from 981 study participants were examined. Five neurocognitive domains were evaluated: motor skills, speed of information processing, attention/working memory, executive function and verbal episodic memory. NCI was examined as binary (presence/absence) and continuous (mean z-score) outcomes against Alcohol Use Disorders Identification Test for Consumption (AUDIT-C) scores using logistic and linear regression models, respectively.

**Results:**

Most participants (96.2%) had undetectable viral loads and 64% were aged >50 years old. Hazardous alcohol consumption was observed in 49.4% of participants and binge drinking in 4.2%. While alcohol consumption frequency and quantity were not associated with NCI, the practice of binge drinking was significantly associated with impaired motor skills and overall neurocognitive function in both binary (odds ratio, OR ≥2.0, *P* <0.05) and continuous (mean z-score difference -0.2 to -0.4, *P* ≤0.01) outcomes. A significant U-shaped distribution of AUDIT-C score was also observed for motor skills and overall neurocognitive function.

**Conclusions:**

In this cohort of PWH with well-controlled HIV infection, NCI was associated with the practice of binge drinking rather than alcohol consumption frequency or quantity. Longitudinal analysis of alcohol consumption and NCI in this population is currently underway.

## Introduction

HIV-associated neurocognitive impairment (NCI) represents a clinical challenge which will grow as people with HIV (PWH) continue to age. In the era of modern and highly efficacious antiretroviral therapy (ART), potentially modifiable factors affecting HIV course such as alcohol consumption merit review.

The combination of hazardous alcohol consumption and HIV appears to have an association with NCI beyond that of alcoholism or HIV as single diagnoses [1]. Among PWH, alcohol consumption is generally higher than that observed among HIV-negative peers [2,3]. Hazardous alcohol consumption has been observed to be associated with impaired health-related quality of life, impaired ART adherence and ART interruption, all of which potentially affect neurocognitive function [4–7]. Furthermore, in PWH of increasing age, previous hazardous consumption appears to maintain an association with NCI [8–11].

NCI severity in PWH is often characterised according to the Frascati criteria and this enables comparison between different patient cohorts [12,13]. An important disadvantage of the Frascati criteria, where NCI requires deficits in two or more neurocognitive domains, is that deficits in a single domain will be missed. Another disadvantage of the Frascati criteria is that a binary approach of NCI versus no NCI will miss more subtle differences in z-score values. Among HIV-negative individuals, alcohol has been reported to cause global NCI but also both focal deficits affecting specific neurocognitive domains [14–16]. When examining associations between hazardous alcohol consumption (for which there is no uniform definition) and NCI, neurocognitive assessment therefore needs to be tailored carefully. If the effects of alcohol consumption on NCI vary in PWH over time, as has been described among HIV-negative individuals [14], this will be detected only if a detailed neuropsychological assessment is performed at baseline.

To date, there are relatively few studies on the association between alcohol consumption and NCI in the modern ART era (post-2013) beyond prevalence studies [3]. It is unclear whether the effects of alcohol consumption on neurocognitive function are still compounded by HIV when infection is well-controlled. We examined the association between NCI and alcohol consumption among persons enrolled in a large Swiss cohort set up specifically to examine NCI, the Neurocognitive Assessment in the Metabolic and Aging Cohort (NAMACO) study.

## Materials and methods

### Setting and participants

The NAMACO study is a prospective, longitudinal, multicentre and multilingual (French, German and Italian) study of patients nested within the Swiss HIV Cohort Study (SHCS) [17]. Inclusion criteria at enrolment into the NAMACO study were age ≥45 years old, HIV-positive status, enrolment in the SHCS and engagement in care at one of seven SHCS hospital centres (five university-affiliated and two cantonal) (Basel, Bern, Geneva, Lausanne, Zurich, Lugano and St-Gallen). Insufficient oral fluency in the local language to enable neuropsychological testing was the only exclusion criterion [18]. NAMACO participants undergo standardized neurocognitive assessment at inclusion (baseline: between 1^st^ May 2013 and 30^th^ November 2016) and then at two (2016–2018) and four years (2018–2020) from inclusion. The current study examined the baseline data set of 981 NAMACO study participants.

#### Ethics approval and consent to participate

The ethics committees of each cantonal hospital centre (Ethikkommission Nordwest- und Zentralschweiz EKNZ in Basel, Kantonale Ethikkommission Bern in Bern, Commission Cantonale d’Ethique de la Recherche sur l’être humain in Geneva, Commission cantonale d’éthique de la recherche sur l’être humain in Lausanne, Ethikkommission Tessin in Lugano, Ethikkommission Ostschweiz EKOS in St-Gallen and Ethikkommission Zürich in Zurich) approved the NAMACO study protocol. All participants signed informed consent prior to being included.

### Study design

#### Neurocognitive assessment

Neurocognitive assessment in the NAMACO study has been described in detail elsewhere [18,19]. Briefly, assessments were conducted by trained neuropsychologists using a neuropsychological test battery which covered five neurocognitive domains: motor skills, speed of information processing, attention/working memory, executive function and verbal episodic memory. The test battery was based on that used in the International Network for Strategic Initiatives in Global HIV Trials (INSIGHT) and the Strategic Timing of AntiRetroviral Treatment START study group [13]. Each neuropsychological test employed was specific to one of the five neurocognitive domains to avoid unfair weighting of tests due to multiple use (S1 File). The raw score for each neuropsychological test was converted to a demographically adjusted standard score (z-score). NCI was defined according to Frascati criteria as having impairment in at least two out of the five neurocognitive domains [12]. Degrees of NCI were defined as follows: asymptomatic impairment was defined as a decrease of ≥1.0 standard deviation (SD) below normative data in ≥2 neurocognitive domains without functional impairment; mild neurocognitive disorder was defined as a decrease of ≥1.0 SD in ≥2 neurocognitive domains with functional impairment and HIV-associated dementia was defined as a decrease ≥2.0 SD in ≥2 neurocognitive domains with functional impairment [12]. Functional impairment was assessed using Lawton’s Instrumental Activities of Daily Living (IADL) [20,21] and three supplementary questions on work quality and relatives’ observations regarding cognitive decline based on the Patients’ Assessment of Own Functioning Inventory questionnaire (S2 File) [18,19].

#### Alcohol consumption

Alcohol consumption was quantified using the three-item Alcohol Use Disorders Identification Test for Consumption (AUDIT-C) [22,23]. AUDIT-C examines: 1) the *frequency* of alcohol consumed, 2) the *quantity* (where one alcoholic drink approximates to 10g of alcohol) and 3) the practice of *binge drinking* (>six alcoholic drinks in a single sitting). Each question scores between 0 and 4 points and scores are combined to give a total score of between zero and 12. Hazardous drinking in this study was defined as AUDIT-C scores ≥4 in men and ≥3 in women [3,24]. AUDIT-C scores were examined by category (frequency, quantity and binge) and by total score. Data on alcohol consumption among participants in the SHCS are collected at twice-yearly standardized infectious diseases clinic visits. For this study, AUDIT-C scores were obtained from the SHCS database using the extraction from just prior to the neuropsychological assessment. The median interval between AUDIT-C score collection and neuropsychological assessment was 36.5 days (IQR 13.5;73). For participants with an AUDIT-C score of zero, we examined SHCS data and local medical records to determine whether abstinence was related to prior hazardous alcohol consumption or to life-long abstinence. Patient demographic factors (age, sex, ethnicity, education level), HIV history and data on drug use, injecting and non-injecting, were also obtained from the SHCS database.

#### Statistical analysis

Descriptive analyses are presented as mean (SD) for symmetric continuous variables, median (interquartile range, IQR) for asymmetric continuous variables and as percentages for categorical variables. We defined binary (presence/absence of NCI according to Frascati criteria as described above) and continuous (mean z-score) outcomes for each of the five neurocognitive domains and for overall neurocognitive function. The continuous outcome for each neurocognitive domain was defined as the mean of the z-scores of the neuropsychological tests used. Overall neurocognitive function was the mean of the z-score means of the five neurocognitive domains [18].

The association between the variables defining alcohol consumption (frequency, quantity, binge and total AUDIT-C score) and NCI was examined using logistic regression models for the binary NCI outcome and linear regression models for the continuous outcome, adjusted for sociodemographic variables (age, sex, origin, education level and drug use). Given that this is a multi-centre study, we performed a sensitivity analysis adjusting for study centre and observed that such adjustment had minimal effects on the associations we report as significant.

AUDIT-C score was analysed in four models: frequency, quantity, binge drinking and total score. The first three models used binary outcomes where frequency ≥2–4 times a week was coded as 1 and lower frequencies were coded as zero, quantity ≥3–4 drinks was coded as 1 while lower quantities were coded as zero, and binge drinking > once a week was coded as one and ≤ once a week was coded as zero. For the total AUDIT-C score model, the combined scores for frequency, quantity and binge (giving a score of zero to twelve) were taken as continuous covariables. The association between total AUDIT-C score and NCI (binary and continuous outcomes) was examined introducing into the model the total AUDIT-C score (total score) and the total AUDIT-C score squared (total score^2^), the latter to test for a quadratic association between AUDIT-C score and NCI.

Statistical analyses were conducted using R Development Core Team version 3.2 2015 (R Foundation for Statistical Computing, Vienna, Austria; www.R-project.org).

## Results

### NAMACO participants

The demographic details of the 981 NAMACO study participants have been described elsewhere [25]. Briefly, 782 (79.7%) were male, 899 (91.7%) Caucasian and 627 participants (63.9%) were aged >50 years old (mean age 54.5±7.5 years). At baseline, most participants (942, 96.2%) had viral loads <50 copies/ml; median CD4+ T cell count was 634 cells/mm^3^ (IQR 468;814) and median nadir CD4+ count was 180 cells/mm^3^ (IQR 74;270) (Table 1).

**Table 1:** Characteristics of patients participating in the NAMACO study.

	Values
Age, mean (SD)	54.5 (7.5)
Male sex, n (%)	782 (79.7)
Caucasian, n (%)	899 (91.7)
Years of education, mean (SD)	13 (2.8)
**Likely mode of HIV acquisition**	
Men who have sex with men, n (%)	506 (51.6)
Heterosexual, n (%)	325 (33.1)
IDU, n (%)	118 (12.0)
Other/unknown, n (%)	32 (3.3)
**Alcohol, tobacco and drug consumption:**	
≥3 drinks during a typical day, n (%)	20 (2.5)
≥1 binge per month, n (%)	63 (7.9)
Cigarette smoking, n (%)	319 (36.7)
History of previous IDU, n (%)	137 (14.0)
Current non-IDU use:	
Current cannabis use, n (%)	103 (10.5)
Current cocaine use, n (%)	16 (1.6)
**HIV parameters:**	
HIV VL < 50 copies/mm^3^, n (%)	942 (96.2)
Current CD4 count, median [IQR]	634 [468–814]
Nadir CD4 count, median [IQR]	180 [74–270]
On ART, n (%)	959 (97.8)
ART duration, years, median [IQR]	12.7 [6.5–18]
**Neurocognitive function (Frascati criteria):**	
Normal neurocognitive function	591 (60.2%)
Asymptomatic neurocognitive impairment	249 (25.4%)
Mild neurocognitive disorder	8 (0.8%)
HIV-associated dementia	6 (0.6%)
Other	127 (13.0%)

Abbreviations: SD, standard deviation; IDU, injecting drug use; VL, viral load; IQR, interquartile ratio; ART, antiretroviral therapy.

### Alcohol consumption

**Fig 1:**
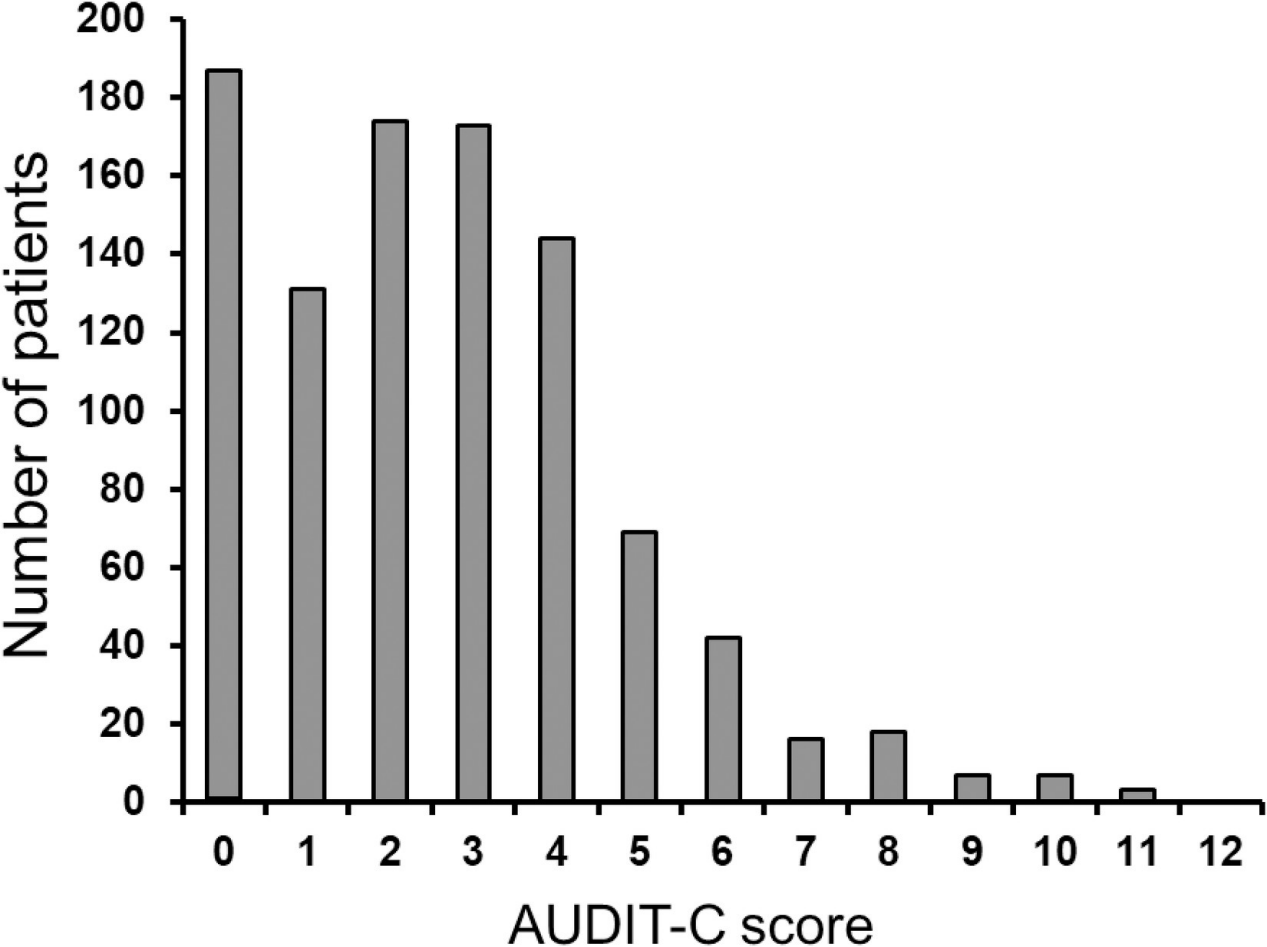
Distribution of total AUDIT-C scores among 981 study participants. Total AUDIT-C score is the sum of the three scores for frequency, quantity and binge drinking. Abbreviations: IDU, injecting drug use.

**Fig 2:**
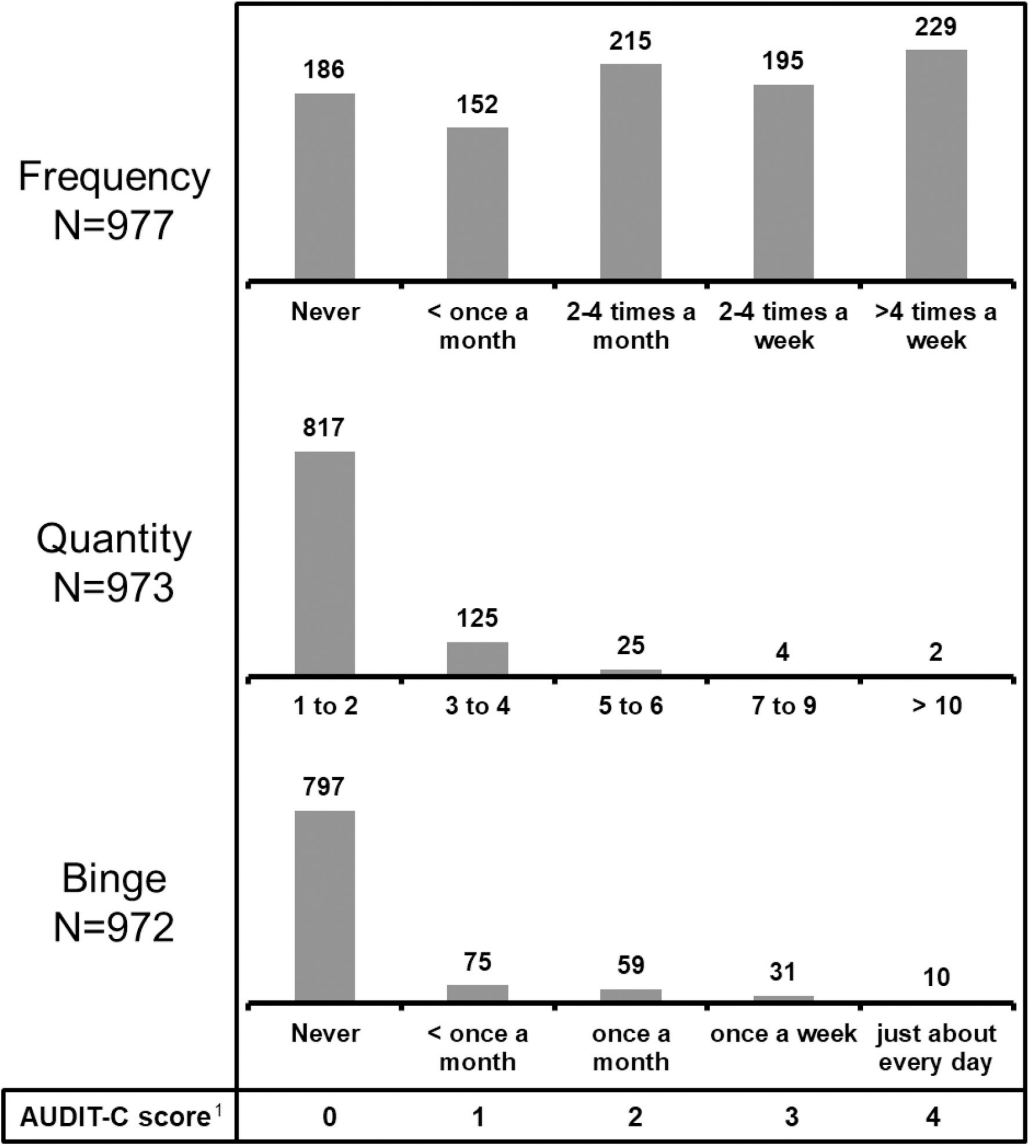
Distribution of alcohol consumption frequency and quantity and binge drinking among 981 study participants. The numbers above each bar in the three bar plots represent the number of participants with each score. ^1^The numbers 0 to 4 are the scores assigned to each possible response in the AUDIT-C score questionnaire. The response options are written below each bar for the three categories of frequency, quantity and binge. Taking the category ‘frequency’ as an example, ‘never’ scores 0, while ‘>4 times a week’ scores 4.

Complete AUDIT-C score data were available for 970 participants (98.9%). Median AUDIT-C score was 2 (IQR 1;4). Hazardous drinking was observed in 479/970 participants (49.4%): 422/774 men (54.5%) with AUDIT-C scores ≥4 and 57/196 women (29.1%) with AUDIT-scores ≥3. Fig 1 shows the distribution of AUDIT-C scores and Fig 2 shows the distribution of the three AUDIT-C score categories of frequency, quantity and binge.

Of 186 participants (19.2%) with an AUDIT-C score of zero, 86 (46.2%) had a history of life-long abstinence prior to NAMACO study inclusion, 62 participants (33%) had a history of alcohol consumption with binge scores ≤1 and 38 (20.4%) had binge scores >1 and/or medical record documentation of prior hazardous consumption.

#### Binary outcome (presence/absence of NCI)

Alcohol consumption was examined first taking all 970 participants with complete AUDIT-C score data and then excluding the 38 participants with AUDIT-C scores of zero at NAMACO study inclusion who had previous histories of hazardous alcohol consumption. Alcohol consumption frequency and quantity, when examined as single variables, were not significantly associated with NCI (Table 2). Taking all participants, binge drinking was significantly associated with impaired motor skills (OR 2.4, *P* = 0.01) and speed of information processing (OR 2.2, *P* = 0.02) (Table 2). Excluding participants with AUDIT-C scores of zero but with previous hazardous alcohol consumption extended the binge drinking association to include overall neurocognitive function (OR 2.0, *P* = 0.04).

**Table 2:** Associations between alcohol consumption and neurocognitive function.

**A Binary Outcome**	Motor skills		Speed of information processing		Attention/working memory		Executive function		Verbal episodic memory		Overall neurocognitive function	
	OR	*P-*value	OR	*P-*value	OR	*P-*value	OR	*P-*value	OR	*P-*value	OR	*P-*value
Frequency	0.93	0.60	1.03	0.86	0.70	0.02	0.86	0.38	1.06	0.77	0.89	0.44
Quantity	2.56	0.32	1.68	0.55	0.00	0.97	0.46	0.51	0.73	0.78	1.16	0.87
Binge drinking	2.42	**0.01**	2.15	**0.02**	0.91	0.80	1.23	0.59	0.79	0.61	1.90	0.06*
Total score	0.88	0.12	0.94	0.48	0.88	0.14	0.87	0.14	1.04	0.69	0.82	0.02
(Total score)^2^	1.02	0.04	1.01	0.30	1.01	0.51	1.02	0.20	0.99	0.44	1.02	0.03
**B Continuous Outcome**	Motor skills		Speed of information processing		Attention/working memory		Executive function		Verbal episodic memory		Overall neurocognitive function	
	Effect	*P-*value	Effect	*P-*value	Effect	*P-*value	Effect	*P-*value	Effect	*P-*value	Effect	*P-*value
Frequency	0.00	0.96	0.01	0.92	0.16	0.001	0.04	0.33	-0.02	0.69	0.03	0.33
Quantity	-0.39	0.22	-0.02	0.95	0.50	0.10	0.13	0.57	0.03	0.92	0.04	0.83
Binge drinking	-0.39	**0.001**	-0.24	0.08	-0.03	0.79	-0.21	**0.02**	-0.12	0.30	-0.20	**0.01**
Total score	0.04	0.18	0.04	0.29	0.05	0.09	0.05	0.02	-0.01	0.72	0.03	0.07
(Total score)^2^	-0.01	0.03	0.00	0.32	0.00	0.53	0.01	0.02	0.00	0.58	-0.00	0.90

Neurocognitive function, examined by neurocognitive domain and overall, was examined in four models of alcohol consumption derived from the AUDIT-C score: frequency, quantity, binge drinking and total AUDIT-C score. All models were adjusted for sociodemographic factors and drug use. Effect in the continuous model refers to mean z-score difference. *P*-values in bold text indicate significant associations with *impaired* neurocognitive function.

Abbreviations: OR, odds ratio.

*In the analysis excluding participants with AUDIT-C scores of zero who had previous hazardous alcohol consumption, the odds ratio was 2.0, P = 0.04.

**Fig 3:**
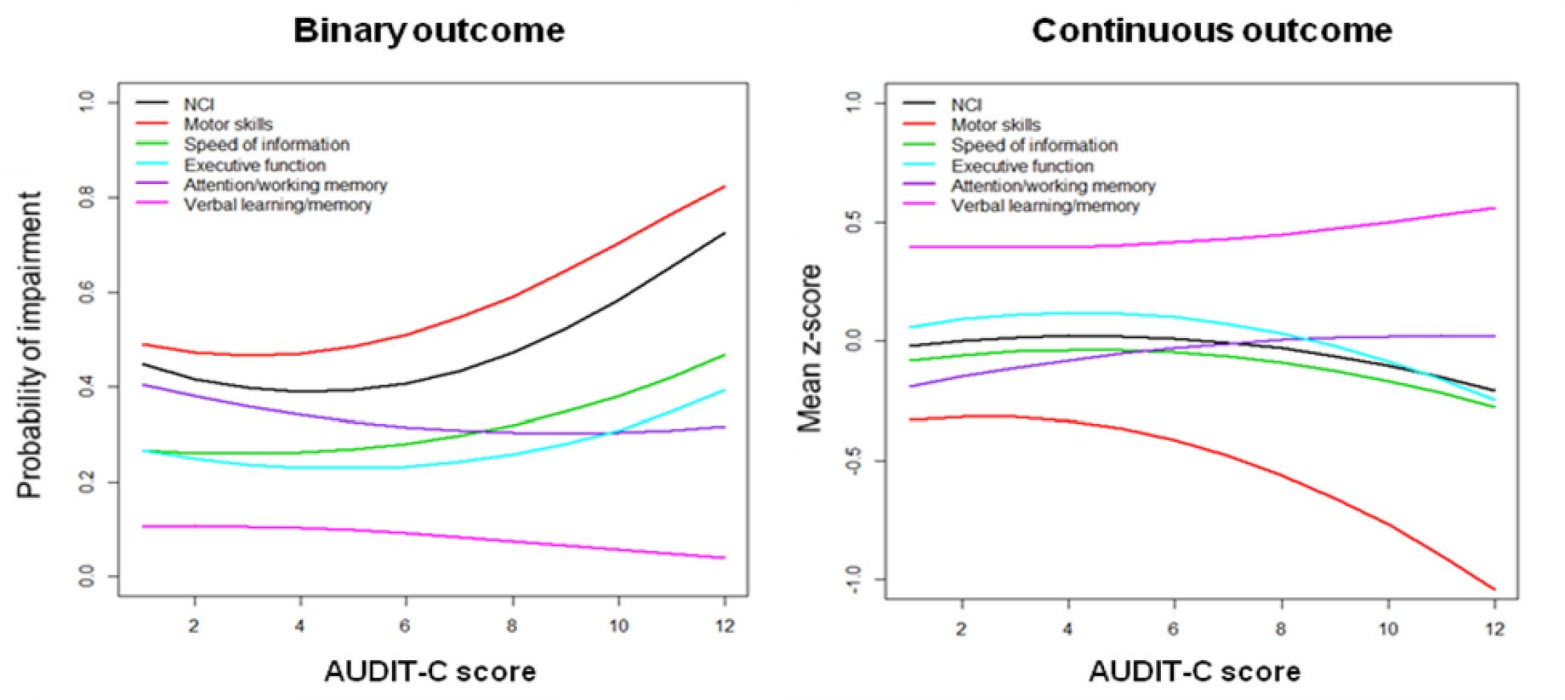
Probability of NCI (binary outcome) and mean z-score (continuous outcome), for each neurocognitive domain and overall NCI, as a function of the AUDIT-C score. NCI is adjusted for sociodemographic variables and drug use, and evaluated at reference values of binary covariates (male, Caucasian and no drug use) and at mean values of age and education level.

We observed a significant quadratic association between AUDIT-C score and motor skills and overall neurocognitive function. As the AUDIT-C score increased from zero to 12, we observed an initial increase (for low scores) in the risk of NCI followed by a significant increase for higher scores (linear AUDIT-C score OR 0.88, *P* = 0.115; quadratic AUDIT-C score OR 1.02, *P* = 0.03 for motor skills; linear AUDIT-C score OR 0.82, *P* = 0.02; quadratic AUDIT-C score OR 1.02, *P* = 0.025 for overall neurocognitive function) (Fig 3).

#### Continuous outcome (mean z-score)

Again, analyses were performed taking all participants and then after excluding participants with AUDIT-C scores of zero but with previous hazardous alcohol consumption. As with the binary outcome, alcohol consumption frequency and quantity were not significantly associated with NCI (Table 2). Binge drinking was significantly associated with impaired motor skills (mean z-score difference -0.39, *P* = 0.001), executive function (mean z-score difference -0.21, *P* = 0.02) and overall neurocognitive function (mean z-score difference -0.20, *P* = 0.01) (Table 2).

We observed a significant quadratic association between AUDIT-C score and motor skills and executive function z-scores. As the AUDIT-C score increased from zero, we observed an initial reduction (for low scores) of the mean z-score followed by a significant reduction for higher scores (linear AUDIT-C score z-score difference 0.039, *P* = 0.184; quadratic AUDIT-C score z-score difference -0.008, *P* = 0.029 for motor skills; linear AUDIT-C score z-score difference 0.051, P = 0.016; quadratic AUDIT-C score z-score difference -0.006, *P* = 0.02 for executive function) (Fig 3).

Repeating the continuous outcome analyses excluding participants with AUDIT-C scores of zero but previous hazardous consumption did not change NCI associations. For both binary and continuous outcomes, associations with drug use and NCI were minor and inconsistent across models.

## Discussion

In this large Swiss population of PWH aged ≥45 years old with well-controlled HIV infection, we observed that hazardous alcohol consumption was more frequently reported among men than women. Whether NCI was considered as a binary (presence/absence) or continuous (mean z-score) outcome, we observed an association between binge drinking and impaired motor skills and overall neurocognitive function. No such associations with NCI were observed with alcohol consumption frequency or quantity. Finally, we observed a U-shaped distribution in which participants with AUDIT-C scores of zero had worse neurocognitive function compared to those with low and moderate alcohol consumption.

This is the first study in Switzerland to examine the association between alcohol consumption and neurocognitive function in any patient group. Among our participants, hazardous alcohol consumption, according to the definition used in our study, was observed in 49.4%. Comparing our findings to another study performed in the modern ART era among 8567 PWH from seven US sites, we observed lower binge drinking levels [3]. Using the binge definition in this US study (less than monthly and more frequently), we observed binge drinking among 18% of participants compared to their figure of 34% (the US study did not provide a figure for binge drinking using our definition of at least weekly). However, the patients in the US study were younger than participants in our study, with a mean age of 46 years and 41% aged ≥ 50 years. The fact that we did not identify an association between drug use and NCI might be related to the relatively small proportion of participants who reported drug use, or indeed to underreporting, but is also in keeping with previous studies [25,26].

The possible protective effect of low to moderate alcohol consumption has been described among HIV-negative persons. In a review of 143 papers examining cognitive risk, light to moderate alcohol consumption appeared to reduce the risk of cognitive decline in older subjects [16]. The small increase in NCI we observed with no alcohol consumption could not be explained by abstinence related to prior hazardous consumption, as excluding the participants in this category did not alter the U-shaped distribution. The neurocognitive profile of these participants at the two-year NAMACO study follow-up is undergoing analysis to examine this association.

Adverse effects of HIV and alcoholism on specific neurocognitive domains have been described. Fama and co-authors described two single-centre studies exploring the effect of HIV, alcoholism and HIV plus alcoholism on neurocognitive function against neurocognitive function among control participants with neither HIV nor alcoholism [27,28]. The number of participants was smaller than in our study, with 36–42 participants per group, and the participants were younger, mean age 40–45 years old at baseline (compared to 54.5 years in our study). This group observed that HIV and alcoholism were associated with decreased scores on executive function and episodic memory compared to control participants. As well as marked differences in sample size and participant age, the two studies reported by Fama and co-authors differed from our study in several ways. First, this group defined alcoholism as meeting DSM-IV criteria for alcohol dependence within three years prior to baseline study entry rather than by using the AUDIT-C score. Second, they examined two neurocognitive domains, first working and episodic memory [27] and then executive function and episodic memory [28], rather than the range of neurocognitive domains we used. Third, they used a computerised neuropsychological test battery, the MicroCog; our study used different neuropsychological tests and the assessment was performed by clinical neuropsychologists. The association we observed in our study between alcohol consumption and impairment in motor skills and overall neurocognitive function may therefore be explained by both participant demographic and methodological factors.

What our study adds to the effect of alcohol on NCI is the effect of binge drinking as opposed to hazardous alcohol consumption *per se*. Binge drinking has been described in the context of liver disease. In a population-based study of 6366 adults aged ≥30 years old (HIV status not reported) set in Finland, weekly and monthly binge drinking was associated with an increased risk for liver disease which was independent of average alcohol intake [29]. To our knowledge, ours is the first study to report this association in the context of NCI.

Our study has limitations. All alcohol and drug use data were obtained at medical visits within the SHCS and documented by the patients’ infectious diseases physicians rather than by anonymous questionnaire. As a result, the data may be subject to some recall and/or social desirability bias. AUDIT-C scores were not supplemented with phosphatidylethanol measurements. We did not apply formal diagnostic criteria for substance use but used data on substance type and frequency of use. We did not adjust for smoking. However, smoking was not identified as a factor associated with NCI in our NCI prevalence paper [18]. Another limitation is that the NAMACO study did not include HIV-negative control participants. In this way, even though our study population had excellent HIV control (96.2% with viral loads <50 copies/ml), the associations we present between alcohol consumption and NCI should still be considered in the context of HIV infection. Finally, women made up 20% of NAMACO study participants and fewer women than men described hazardous drinking (29% versus 55%). Even though this left 57 female participants with hazardous drinking, it is possible that we did not observe an effect of female sex on NCI due to insufficient numbers. Against these limitations, we have compared the effect of alcohol consumption in a well-characterised cohort with higher numbers of participants than described to date [27,28]. Our study is novel in that, as well as applying Frascati criteria to examine overall NCI, we examined the effect of alcohol consumption on specific neurocognitive domains, examining z-scores as well as the presence or absence of NCI. A strength of the NAMACO study is that, as recent or previous hazardous drinking and abstention can influence NCI, and as the course of NCI may change over time, our longitudinal analysis will add to the current cross-sectional findings. Our study adds to the Veterans Aging Cohort Study by Williams *et al*., which used the AUDIT-C score to examine the effect of alcohol consumption on HIV disease severity over time [30]. This group examined several health parameters but not neurocognitive function.

### Conclusions

This is the first large study conducted among aging PWH in the modern ART era to examine the association between reported alcohol consumption and impairment in specific neurocognitive domains. We observed that binge drinking, rather than alcohol consumption frequency or quantity, was associated with NCI, both globally and in specific neurocognitive domains. Examining total AUDIT-C score, rather than the binge-drinking component, may therefore risk missing patients at risk of NCI. Our findings could help to inform PWH of the potential hazard of binge drinking in terms of later neurocognitive function. Our neurocognitive assessment at two and four years from this baseline study will contribute to our understanding of the longitudinal association between alcohol consumption and NCI among NAMACO study participants.

## Supporting information

S1 File The five cognitive domains examined and the neuropsychological tests performed in the neurocognitive assessment of study participants. (DOCX)

S2 File Functional assessment questions used in the NAMACO study. (DOCX)

## Abbreviations

ART: antiretroviral therapy
AUDIT-C: Alcohol Use Disorders Identification Test for Consumption
NAMACO: Neurocognitive Assessment in the Metabolic and Aging Cohort
NCI: neurocognitive impairment
PWH: people with HIV
SHCS: Swiss HIV Cohort Study
