# Supplementary material for: Alcohol consumption and neurocognitive deficits in people with well-treated HIV in Switzerland
Source: PLoS One. 2021 Mar 2;16(3):e0246579. doi: 10.1371/journal.pone.0246579 (PMC7924787; doi:10.1371/journal.pone.0246579)
Supplement: S1 File — (DOCX) [file pone.0246579.s001.docx]

**S1 File. The five cognitive domains examined and the neuropsychological tests performed in the neurocognitive assessment of study participants.**

| **Standardised neurocognitive assessment** | |
| --- | --- |
| **Cognitive domain** | **Neuropsychological tests** |
| Motor skills | Finger Tapping (dominant & non-dominant hand)  Grooved Pegboard (dominant & non-dominant hand) |
| Speed of information processing | WAIS-IV, Coding  Colour Trails 1 |
| Attention & working memory | WAIS-IV Digit Span (forward & backward) |
| Executive function | Category Fluency^1^  5-point Figural Fluency  Victoria Stroop (trial 3 and/or 3/1)  Colour Trails 2 |
| Verbal episodic memory | Hopkins Verbal Learning Test – Revised^2^ |

Abbreviations: WAIS, Wechsler Adult Intelligence Scale 4^th^ Edition

^1^ Visits 1 and 3: animals; visit 2: food

^2^ In testing verbal episodic memory, z-scores were calculated for four measurements: total trial 1, 2, 3; delayed recall; percentage retention and recognition discrimination index.
