# Supplementary material for: Alcohol consumption and neurocognitive deficits in people with well-treated HIV in Switzerland
Source: PLoS One. 2021 Mar 2;16(3):e0246579. doi: 10.1371/journal.pone.0246579 (PMC7924787; doi:10.1371/journal.pone.0246579)
Supplement: S2 File — (DOCX) [file pone.0246579.s002.docx]

**IADL Scale (Instrumental Activities of Daily Living)**

**PATIENT ID:__________ DATE OF VISIT: ___________ VISIT N°: ____**

| **A.** | **Ability to use telephone** |  |
| --- | --- | --- |
|  | 1. Operates telephone on own initiative; looks up and dials numbers | 1 |
|  | 1. Dials a few well-known numbers | 1 |
|  | 1. Answers telephone but does not dial | 1 |
|  | 1. Does not use telephone at all | 0 |
| **B.** | **Shopping** |  |
|  | 1. Takes care of all shopping needs independently | 1 |
|  | 1. Shops independently for small purchases | 0 |
|  | 1. Needs to be accompanied on any shopping trip | 0 |
|  | 1. Completely unable to shop | 0 |
| **C.** | **Food preparation** |  |
|  | 1. Plans, prepares, and serves adequate meals independently | 1 |
|  | 1. Prepares adequate meals if supplied with ingredients | 0 |
|  | 1. Heats and serves prepared meals or prepares meals but does not maintain adequate diet | 0 |
|  | 1. Needs to have meals prepared and served | 0 |
| **D.** | **Housekeeping** |  |
|  | 1. Maintains house alone with occasional assistance (heavy work) | 1 |
|  | 1. Performs light daily tasks such as dishwashing, bed making | 1 |
|  | 1. Performs light daily tasks but cannot maintain acceptable level of cleanliness | 1 |
|  | 1. Needs help with all home maintenance tasks | 1 |
|  | 1. Does not participate in any housekeeping tasks | 0 |
| **E.** | **Laundry** |  |
|  | 1. Does personal laundry completely | 1 |
|  | 1. Launders small items, rinses socks, stockings, etc. | 1 |
|  | 1. All laundry must be done by others | 0 |
| **F.** | **Models of transportation** |  |
|  | 1. Travels independently on public transportation or drives own car | 1 |
|  | 1. Arranges own travel via taxi, but does not otherwise use public transportation | 1 |
|  | 1. Travels on public transportation when assisted or accompanied by another | 1 |
|  | 1. Travel limited to taxi or automobile with assistance of another | 0 |
|  | 1. Does not travel at all | 0 |
| **G.** | **Responsibility for own medications** |  |
|  | 1. Is responsible for taking medication in correct dosages at correct time | 1 |
|  | 1. Takes responsibility if medication is prepared in advance in separate dosages | 0 |
|  | 1. Is not capable of dispensing own medication | 0 |
| **H.** | **Ability to handle finances** |  |
|  | 1. Manages financial matters independently (budgets, writes checks, pays rent and bills, goes to bank); collects and keeps track of income | 1 |
|  | 1. Manages day-to-day purchases, but needs help with banking, major purchases, etc | 1 |
|  | 1. Incapable of handling money | 0 |

**Source**: Lawton MP, Brody EM. Assessment of older people: self-maintaining and instrumental activities of daily living. Gerontologist. 1969;9(3):179-186.

**Supplementary questions on job performance**

| **I.** | **Job performance** |  |
| --- | --- | --- |
|  | 1. Unable to perform some aspects of previous job (not due to medical symptoms) | 0 |
| **L.** | **Job performance** |  |
|  | 1. Reduced efficiency or productivity; or more errors or difficulties meeting expectations; or greater effort to perform the same activities | 0 |

**Scoring (TOTAL)**: If the patient receives a score of 0 for at least two of the items above (A-L), then s/he is considered to be functionally impaired.

<https://www.sm.ee/sites/default/files/content-editors/eesmargid_ja_tegevused/Tervis/Ravimid/eacsguidelines_v6.1_nov2012.pdf>

**Source**: Antinori A, Arendt G, Becket JT, et al. Updated research nosology for HIV-associated neurocognitive disorders. *Neurology*. 2007;69(18):1789-99.

**Supplementary question concerning social entourage**

| **M.** | **Entourage** |  |
| --- | --- | --- |
|  | 1. Comments made by entourage (close family, friends, colleagues, etc.) regarding decline in cognitive function | 0 |
